# Dynamics of CH/*n* hydrogen bond networks probed by time-resolved CARS spectroscopy†

**DOI:** 10.1039/d4sc03985h

**Published:** 2024-07-31

**Authors:** Hanlin Zhu, Xinyu Deng, Vladislav V. Yakovlev, Delong Zhang

**Affiliations:** a Zhejiang Key Laboratory of Micro-nano Quantum Chips and Quantum Control, School of Physics, Zhejiang University Hangzhou Zhejiang 310027 China dlzhang@zju.edu.cn; b MOE Frontier Science Center for Brain Science & Brain-Machine Integration, Zhejiang University Hangzhou Zhejiang 310027 China; c Department of Biomedical Engineering, Texas A&M University College Station TX 77843 USA yakovlev@tamu.edu; d Department of Physics and Astronomy, Texas A&M University College Station TX 77843 USA; e Department of Electrical and Computer Engineering, Texas A&M University College Station TX 77843 USA

## Abstract

Hydrogen bond (HB) networks are essential for stabilizing molecular structures in solution and govern the solubility and functionality of molecules in an aqueous environment. HBs are important in biological processes such as enzyme–substrate interactions, protein folding, and DNA replication. However, the exact role of weakly polarized C–H bonds as HB proton donors in solution, such as CH/*n* HBs, remains mostly unknown. Here, we employ a novel approach focusing on vibrational dephasing to investigate the coherence relaxation of induced dipoles in C–H bonds within CH/*n* HB networks, utilizing time-resolved coherent anti-Stokes Raman scattering (T-CARS) spectroscopy. Using a representative binary system of dimethyl sulfoxide (DMSO)–water, known for its C–H backboned HB system (*i.e.*, C–H⋯S), we observed an increase in the dephasing time of the C–H bending mode with increasing water content until a percolation threshold at a 6 : 1 water : DMSO molar ratio, where the trend is reversed. These results provide compelling evidence for the existence of C–H⋯S structures and underscore the presence of a percolation effect, suggesting a critical threshold where long-range connectivity is disputed.

## Abstract 2

Vibrational dephasing spectroscopy reveals a new percolation threshold in CH/*n* hydrogen bond networks.

## Graphical abstract

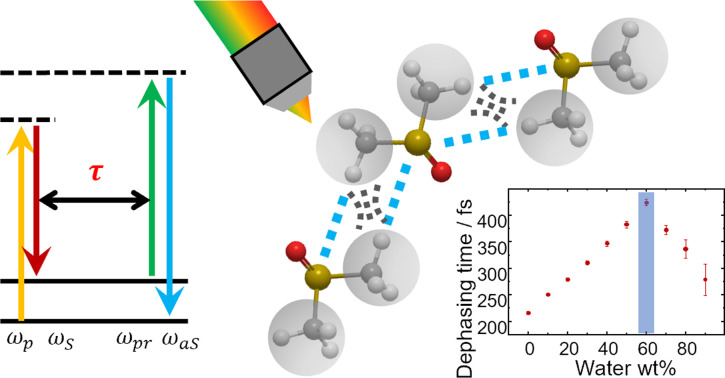

## Introduction

The hydrogen bond (HB) is a delicate, but essential molecular interaction in significant biological systems and is utilized by nature for construction of a unique liquid system with high solubility of polar molecules. The presence of HB networks can influence the physical properties of materials such as the surface tension, melting point, boiling point, and mechanical strength of a material. Overall, HB networks are essential for maintaining the integrity and functionality of biological systems, as well as influencing both the physical and chemical properties of solutions. Recently, a rather controversial topic has attracted significant attention, the existence of a weakly polarized C–H bond as a HB proton donor,^1,2^ such as the CH/*n* HB (*n* refers to lone pair electrons), contrary to the classical requirements for proton donors from few groups of the periodic table, such as HBs composed of O–H or N–H bonds.^3^ CH/*n* HB networks could help maintain a substantial number of three-dimensional structures or conformations in biological systems, such as higher order structures of proteins.

**Fig. 1:**
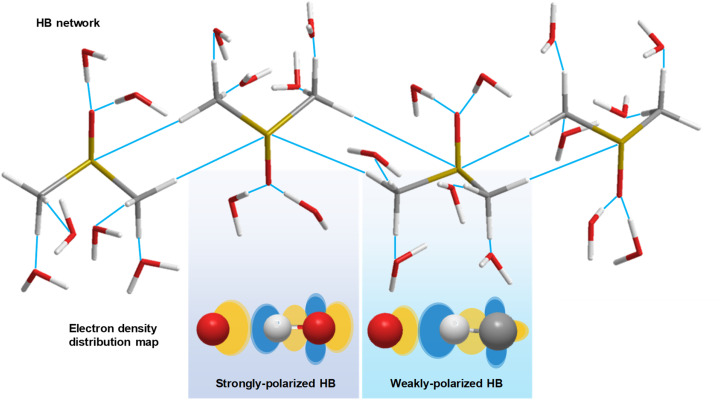
Schematic diagram of the HB network in DMSO–water solution. Strongly polarized and weakly polarized HBs influence the electron density redistribution. White stick (ball): hydrogen atom. Grey stick (ball): carbon atom. Red stick (ball): oxygen atom. Yellow stick: sulfur atom. Orange ellipse: high electron density region. Blue ellipse: low electron density region.

However, collecting evidence of the existence of a CH/*n* HB network in the liquid phase is challenging. For example, nuclear magnetic resonance (NMR) spectroscopy identifies the presence of HB networks through the characterization of proton-mediated scalar (*J*) coupling.^4,5^ Such an experiment, however, is subject to rapid molecular reorganizations and competing interactions in solution, presenting formidable challenges.^6^ Alternatively, infrared (IR) spectroscopy, which is directly related to changes in molecular dipole moment, is capable of revealing strongly and weakly polarized HBs in solution (Fig. 1). The formation of weakly polarized HBs causes a shortening of the C–H bond, detectable *via* IR resonant frequency, *i.e.*, an improper blue-shift.^7^ Furthermore, IR difference spectroscopy amplifies subtle spectral variations, revealing protein properties, such as protonation.^8^ Additionally, Raman spectroscopy, a vibrational modality based on inelastic photon scattering, complements IR spectroscopy by providing detailed insights into molecular vibrations and vibrational energy levels,^9,10^ yet suffers from low signal levels. Collectively, a more capable tool to explore CH/*n* HB networks against the interfering local HB effects is urgently needed.

To better understand the mechanism of formation and the physicochemical nature of HB networks, a versatile molecular system, dimethyl sulfoxide (DMSO)–water solution, is often employed as a model system. Based on this binary system, various properties regarding HB networks have been studied,^11^ including common solvent for drug screening,^12^ biological antifreeze agents,^13^ and chemical catalysis.^14^ The DMSO–water system has unique properties, where its freezing point,^15^ viscosity, and density^16^ reach the extremum at concentrations around 30–40 mol% DMSO. There are a series of theoretical and experimental studies to comprehensively understand such unique behaviours, including the generation of eutectic composition using HBs,^17^*i.e.*, a stable 2 : 1 water : DMSO structure.^18^ Furthermore, at lower concentrations (10–20 mol% DMSO), the DMSO–water system displays unique solvent properties due to micro-environment change. For instance, lower DMSO concentrations (<15 mol%) stabilize proteins by preferential solvation in the hydrophobic enzymatic active region, whereas at higher concentrations, the hydrophobic core connecting DMSO molecules starts to decrease, enhancing protein activities.^19^ Furthermore, mass spectroscopy results reveal that the formation of clusters does not follow a linear relation with its concentration. Specifically, water clusters start to show up only when the DMSO concentration is decreased to below 20 mol%, followed by a sharp increase in water clusters at 10 mol%.^20^ To understand such phase transitions of molecular aggregation in DMSO–water solution, the percolation effect is proposed.^21^ A lattice model has been suggested to depict the percolation cluster, where the hydrophobicity of methyl groups of DMSO is considered a principal factor in cluster generation.^22^ However, experimental evidence remains insufficient to provide a comprehensive understanding of CH/*n* HB networks at the percolation threshold.

It is worth discussing the fundamentals of the percolation threshold. The percolation effect is crucial in the formation of long-range connected clusters, such as in proteins, and provides a pathway for vibrational energy flow between distant functional sites, thereby enabling the regulation of chemical reactions.^23^ Therefore, understanding the percolation threshold is of great importance, leading to various theoretical and experimental investigations. For instance, stochastic processes and molecular dynamics simulations have been developed to estimate the percolation threshold.^19,24^ Moreover, molecular vibrational techniques, such as near-IR^25^ and Fourier transform IR spectroscopies,^26^ have been utilized to observe the resonant frequency shift near the percolation state. However, percolation threshold detection remains underexplored by static molecular vibrational spectroscopy.

Recently, time-resolved vibrational spectroscopy was used to investigate decoherence of molecular vibrations, which provides essential dynamic information from the temporal dimension.^27^ Wong *et al.* measured a maximum orientational time constant at 67 mol% water utilizing the optical heterodyne detected optical Kerr effect, which indicates the concentration of DMSO–water solution with maximum viscosity.^28^ Oh *et al.* detected the orientational relaxation times of polar and nonpolar probe molecules in DMSO–water solution by two-dimensional IR spectroscopy, which suggested a local bulk environment.^29^ Roy *et al.* analysed the simulation results of reorientation time of DMSO–water solution and found a local maximum at 15–20 mol% DMSO,^30^ whose origin is considered to be the percolation effect. Overall, while evidence has emerged for a percolation threshold, the experimental result is still insufficient to illustrate the underlying principles of DMSO–water clusters.

Here, we demonstrate, to the best of our knowledge, the first application of time-resolved vibrational spectroscopy to show its unparalleled sensitivity to investigate CH/*n* HB networks, implemented by the time-resolved coherent anti-Stokes Raman scattering (T-CARS) spectroscopy technique. The principle of T-CARS spectroscopy is introduced elsewhere.^31^ In brief, the selected molecular vibrational mode is coherently excited using a pulse pair, a pump beam and a Stokes beam, whose frequency difference matches the specific resonant frequency. A probe beam with a series of time delays is used to generate a CARS signal which indicates the vibrational dephasing dynamics of the sample.

We discuss the principles of vibrational dephasing to underline its unique sensitivity in investigation of the percolation effect. Transient polarization of samples under laser excitation is depicted using the optical Bloch equation:^32^1
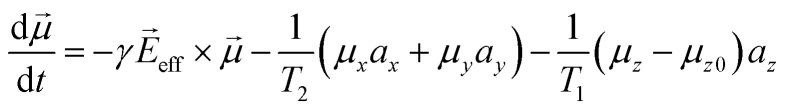
where *μ* is the optical dipole moment, which is the linear addition of the electric dipole moment at the population state (*μ*_*z*_) and superposition state (*μ*_*x*_ and *μ*_*y*_). Notably, *x*, *y*, and *z* are virtual directions. 
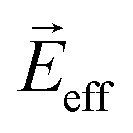
 is an equivalent electric field, which is a linear addition of the electric field of the laser (*E*_*x*_ and *E*_*y*_) and 
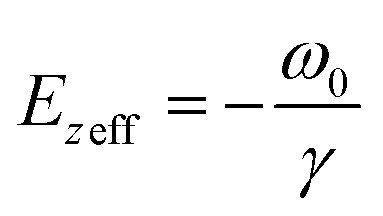
. *ω*_0_ is the frequency of the excited beam, which matches a specific energy level to maintain finite *T*_1_ (lifetime of the population state) and *T*_2_ (dephasing time of the superposition state). *γ* is the ratio of the induced electric dipole and density matrix of a specific energy level. Referring to the Bloch equation of a magnetic dipole, pump and Stokes beams coherently stimulate electric dipoles, which excite the equivalent electric field *E*_*z* eff_. The induced electric dipoles then “rotate”, *i.e.*, a population of superposition states oscillates, driven by *E*_*z* eff_. In the meantime, stochastic perturbation induces dephasing of electric dipoles. After a delay in time, a probe beam arrives and rearranges the polarizations to generate a photon echo. The polarization can be written as:

2 *P* = *χ*^(3)^*E*_pu_*E*_St_*E*_pr_∑e^*iϕ*(*t*)^ = *χ*^(3)^*E*_pu_*E*_St_*E*_pr_e^−*t*/*T*_2_^

where *χ*^(3)^ is the third-order nonlinear susceptibility. *E*_pu_, *E*_St_, and *E*_pr_ are the electric fields of pump, Stokes, and probe beams. The summation includes the ensemble of excited molecules. *ϕ*(*t*) is the phase induced by perturbation. Regarding vibrational modes involving angular momentum, such as bending mode, its dephasing depicts a process of increased randomness in polarization directions. Such non-bound, freely moving bending dipoles are expected to have shorter dephasing time compared with bounded dipoles with restricted alignment of directions.

## Material and methods

### Sample preparation

DMSO (Beijing Wokai Bio-Technology) in a water binary solution system was used. A pipette (Eppendorf, 1 mL) was used to prepare a group of DMSO–water solutions for weight percentages ranging from 10–100% with a 10 wt% gradient. The samples were stored at room temperature.

### T-CARS setup

**Fig. 2:**
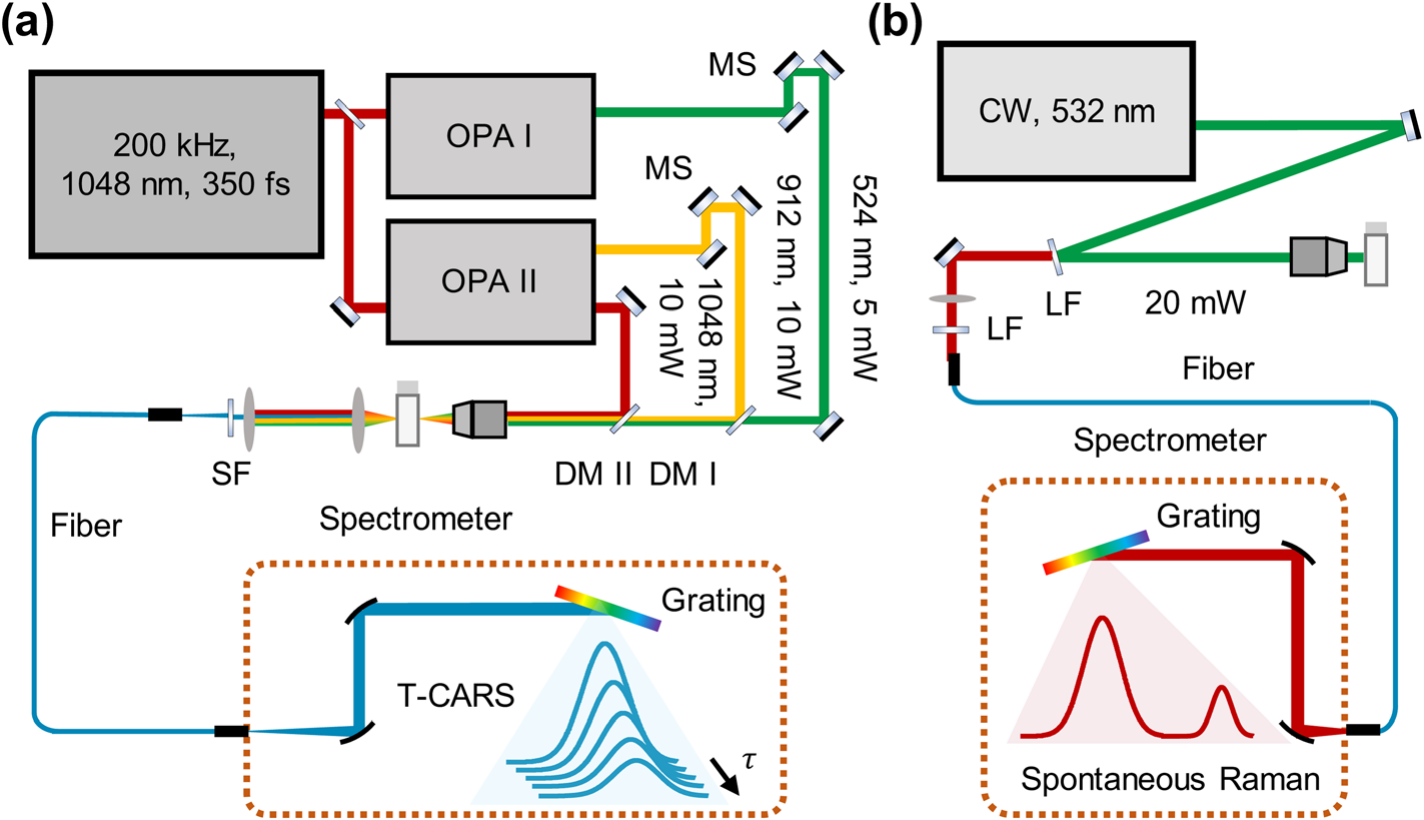
Raman spectroscopic platform. (a) T-CARS setup. OPA: optical parametric amplifiers. Red line: Stokes beam. Orange line: pump beam. Green line: probe beam. MS: moving stage. DM: dichroic mirror. SF: short-pass filter. (b) Spontaneous Raman setup. LF: long-pass filter.

A sketch of the T-CARS spectroscopy setup is depicted in Fig. 2a. The details of instruments are provided elsewhere.^31^ We list the main parameters of the experiments in this section. The fs pump beam is generated using a femtosecond infrared laser (SPIRIT 1040-16-30-HE, MKS instruments). OPA I and OPA II are two optical parametric amplifiers (Orpheus-HP, Spectra-Physics). The pulse duration is 350 fs. The repetition rate is 200 kHz. DM I (DMSP0550, Thorlabs) and DM II (DMSP1000, Thorlabs) are dichroic mirrors used to combine excitation beams. The sample is held in a cuvette. The material of the cuvette is glass. An objective lens (N. A. 0.4, Zolix) is used to focus the beams. SF is a short-pass filter (FESH0500, Thorlabs) used to filter the excitation beams. A fibre (UV-600 μm, Shuchuangzhineng Technology) is used to transport the CARS signal to a spectrometer (Shamrock 750, Andor Technology). The spectrometer acquires the CARS spectra at each time delay of the probe pulse. The grating is 1200 lp/mm and blaze wavelength is 500 nm. An electron-multiplying charge-coupled device (EMCCD, Newton, Andor Technology) is used to measure the CARS signal. The spectral wavelengths of Stokes, pump, and probe beams are 1048 nm, 912 nm, and 524 nm and the spectral bandwidths are 35 cm^−1^, 86 cm^−1^, and 55 cm^−1^ (Fig. S1a†). The frequency difference between the pump and Stokes beams covers the resonant frequency of C–H bending mode (1417 cm^−1^). Energy per pulse of each beam is 50 nJ, 50 nJ, and 25 nJ.

We measured the four-wave-mixing (FWM) signal of water to indicate the chirp effect of the excitation beams (Fig. S1b†). The temporal profile is Gaussian and the full-width-half-maximum (FWHM) is 397 fs (Fig. S1c†). Considering the initial pulse width of the pump beam (350 fs) and the excitation conditions sustained, the chirp effect is not considered in experiments. The vibrational dephasing time of water–DMSO solution is estimated from exponential fitting of the T-CARS signal at each time point, defined as the 1/*e* decay constant. Error bars were evaluated from the standard deviation of fitting. The difference spectra are coloured using the Roma colour map.^33^

### Spontaneous Raman spectroscopy setup

The spontaneous Raman setup is aligned in a backward detection scheme (Fig. 2b). The excitation beam is generated from a solid-state laser at 532 nm (MW-GLN-532, Changchun Femtosecond Technology). The power of the excitation beam is 20 mW. An objective lens (N. A. 0.4, Zolix) is used to focus the beams. A long-pass filter (FELH0550, Thorlabs) is used to separate the excitation beam and Raman scattering. After another long-pass filter (FELH0550, Thorlabs), Raman scattering is directed to a fibre (UV-600 μm, Shuchuangzhineng Technology) and sent into a spectrometer (Shamrock 500i, Andor Technology) with an EMCCD (Newton, Andor Technology). The acquisition time for each spectrum is 5 s. The electron multiplier gain is 100 with an adjustment range of 0–255. The spontaneous Raman spectra were normalized by the maximum intensity of the C–H stretching mode at 2914 cm^−1^ to eliminate variations in excitation conditions.

## Results and discussion

**Fig. 3:**
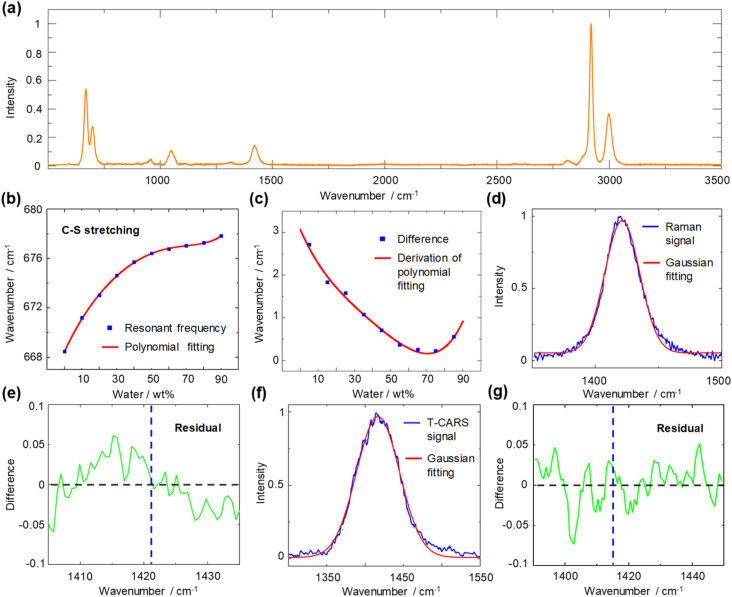
Spontaneous Raman spectroscopy spectra of DMSO–water solution. (a) Spontaneous Raman spectrum of pure DMSO. (b) Blue rectangle: resonant frequency of C–S symmetric stretching mode plotted against the water concentration in weight percent. Red line: fitting. (c) Blue rectangle: difference in resonant frequency. Red line: derivation of polynomial fitting. (d) Raman signal of C–H bending mode. Unimodal Gaussian fitting deviates from the peak. (e) Residual curve of the Raman signal and its Gaussian fit. Blue dotted line: resonant frequency (1421 cm^−1^) by Gaussian fitting. (f) T-CARS signal of C–H bending mode at 1.5 ps time delay. Unimodal Gaussian fitting conforms to the peak. (g) Residual curve of the T-CARS signal and its Gaussian fit. Blue dotted line: resonant frequency (1417 cm^−1^) by Gaussian fitting.

For the DMSO–water system, the sulfur atom of the DMSO molecule has lone pair electrons, which can serve as an *n* HB acceptor^34,35^ and enable connection between DMSO molecules by the CH/*n* HB network. We first measured spontaneous Raman spectra to illustrate the CH/*n* HB network of the DMSO–water cluster (Fig. 3). Considering that the sulfur atom provides electrostatic interaction^36^ on the hydrogen atom to enable the construction of a CH/*n* HB and CHS HB this is one of the strongest non-covalent interactions.^37^ It is interesting to see how the resonant frequency changes with varying solvent concentrations. The measurement of resonant frequency of C–S stretching mode (668 cm^−1^) across varying water concentrations is shown in Fig. 3b (bimodal Gaussian fitting details are presented in Fig. S2†). A 5th order polynomial is used to fit the change in resonant frequency. The resonant frequency is found to be blue-shifted with an increasing water concentration, which can be explained by the involvement of the C–S bond in the CH/*n* HB formation. We further calculated the differences in resonant frequencies between consecutive concentrations and found a decrease in these differences until around ∼70 wt%, where an increase was noted. Subsequent derivation of polynomial fitting reveals the turning point to be at 70 wt% water (Fig. 3c).

To indicate the resonant frequency change of a proton donor, we investigated the spontaneous Raman spectra of C–H bending mode at 1417 cm^−1^ (Fig. S3†). Interestingly, the expected blue shift does not appear; instead, the resonant frequency varies within only 3 cm^−1^. By examining the spectral profiles of the C–H bending mode in DMSO (Fig. 3d), a subtle deviation from a perfectly symmetric peak is observed. The residual curve shows the existence of deviations at 1410–1420 cm^−1^ and 1425–1435 cm^−1^ (Fig. 3e). Such observation agrees with previous assignment of DMSO peaks at 1426 cm^−1^ and 1417 cm^−1^ by Martens *et al.*^38^ However, further analysis of such spectral deviation to pinpoint the underlying mechanisms of the CH/*n* HB network is beyond the current spontaneous Raman system, as the variations in the intensity ratio of these peaks obscure the real resonant frequency and spectral bandwidth change. Thus, a dynamic detection technique is necessary to unravel the peak of interest through the temporal dimension. We therefore implement T-CARS spectroscopy to identify the overlapped peak. As shown in Fig. 3f, the non-resonant background-free T-CARS signal at 1.5 ps time delay shows good agreement with Gaussian fitting. The residual curve exhibits evenly distributed errors, indicating good measurement of the target peak with a longer dephasing time (Fig. 3g).

**Fig. 4:**
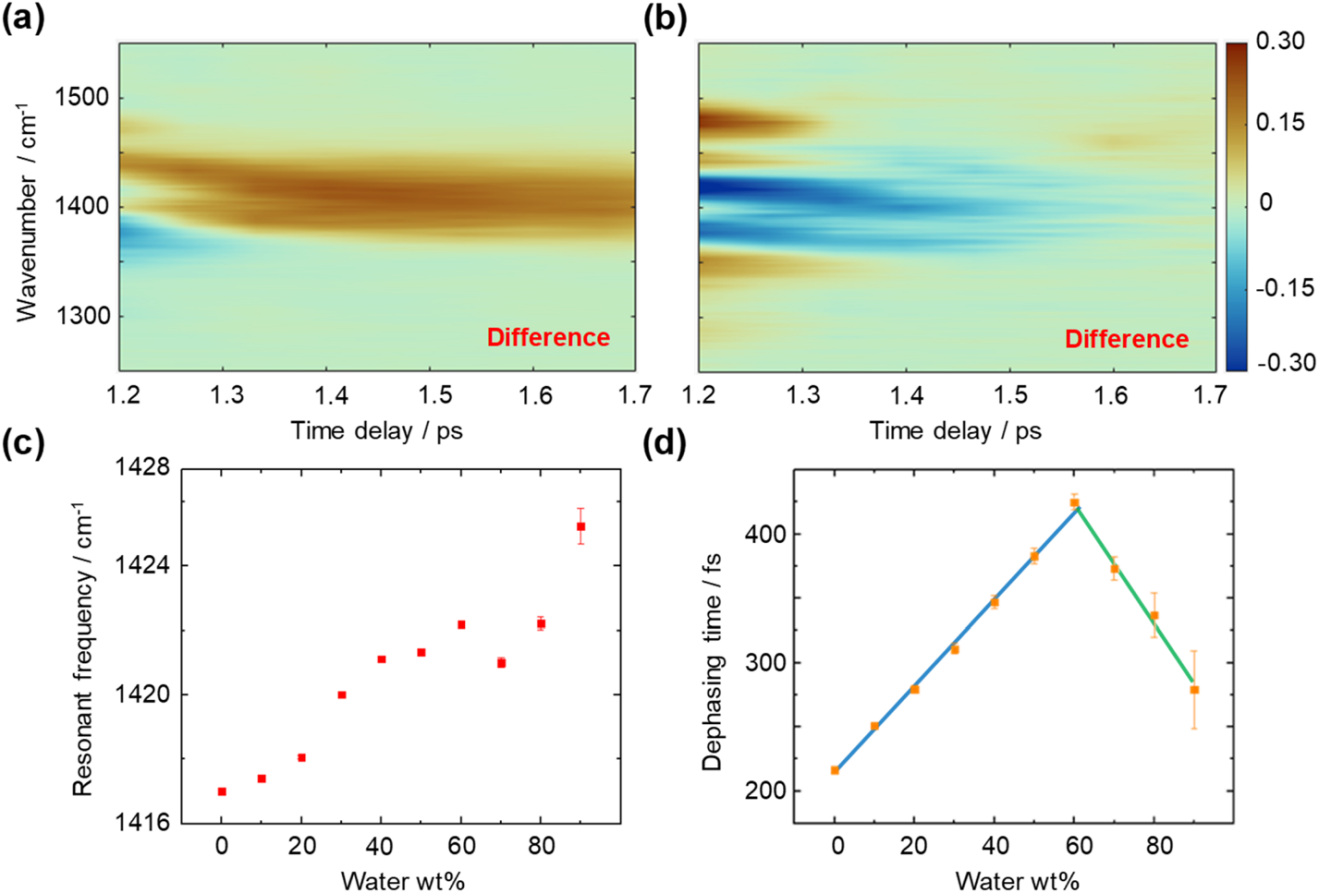
T-CARS spectra of the C–H bending mode in DMSO–water solution. (a) Difference T-CARS spectrum of 60–0 wt% water. (b) Difference T-CARS spectrum of 90–60 wt% water. (c) Resonant frequency of the C–H bending mode at different DMSO concentrations. (d) Molecular vibrational dephasing time of the C–H bending mode at different DMSO concentrations. Blue line: linear fit of the dephasing time at 0–60 wt% water. Green line: linear fit of the dephasing time at 60–90 wt% water.

We then measured the time- and frequency-resolved two-dimensional T-CARS spectra of DMSO with varying concentrations (Fig. 4). To illustrate the contrasting trends of the vibrational dephasing process involving the CH/*n* HB network, difference spectra were calculated (60–0 wt% in Fig. 4a and 90–60 wt% in Fig. 4b). An overall positive difference is observed in the 2D spectrum in Fig. 4a and an overall negative spectrum in Fig. 4b, suggesting a longer dephasing time of 60 wt% than other concentrations. Meanwhile, a negative side peak is found at 1375 cm^−1^ in Fig. 4a while a positive side peak is shown at 1475 cm^−1^ in Fig. 4b, which is caused by the shift of resonant frequency at different concentrations. We quantitively analysed the T-CARS detection results, including resonant frequency (Fig. 4c) and molecular vibrational dephasing time (Fig. 4d) to understand the CH/*n* HB network of DMSO–water solution (processing details in Fig. S4†). With the increment of water content, an improper blue shift of the C–H bending mode is found, which is consistent with theory and experiments (Fig. 4c).^39,40^ Additionally, the vibrational dephasing time of the C–H bending mode is found to increase with increasing water content up to 60 wt% water, which can be attributed to the generation of HBs that limits the motion and decreases the energy consumption of bending mode.

Notably, a turning point at around ∼60 wt% water is found, where the dephasing time starts to decrease. Linear fitting of each segment shows an intersection at 61 wt% water (Fig. 4d), which equals a 6.5 : 1 water : DMSO molar ratio. This phenomenon can be explained using the Bethe lattice model, an infinite tree without loops, which is in accordance with the percolation-driven structure without intra-molecular HBs as loops in DMSO–water solution. Here, the coordination number *z* = 8 for the DMSO molecule and the percolation threshold *P*_c_ = 1/(*z* − 1) indicate a molar ratio of 6 : 1,^41^ which is consistent with the above observation. The ∼3 wt% deviation is considered a finite effect of the nonideal Bethe lattice.

**Fig. 5:**
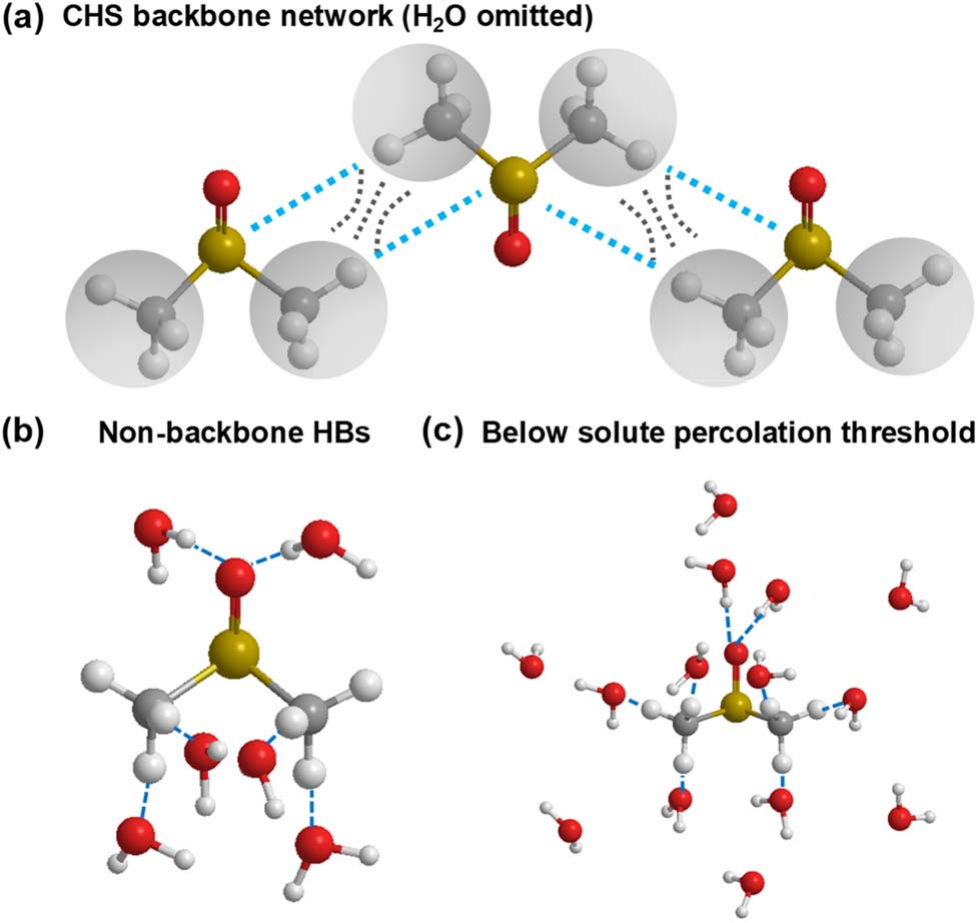
CHS percolation model. (a) DMSO cluster with long-range connectivity at the percolation threshold (6 : 1 water : DMSO). H_2_O is omitted for visual clarity. (b) HB interaction between DMSO and water molecules at the percolation threshold. (c) DMSO–water structure with excess water shells at low DMSO concentrations (<11 mol%). Red ball: oxygen atom. Yellow ball: sulfur atom. White ball: hydrogen atom. Grey ball: carbon atom. Grey circle: methyl. Black dotted line set: hydrophobic interaction between methyl groups. Light blue dotted line: intermolecular HB between sulfur and hydrogen atoms. Blue dotted line: intermolecular HB between oxygen and hydrogen atoms.

The observed behaviours at low DMSO concentrations necessitate a more detailed analysis. Here, we propose a CHS percolation model, in which a 6 : 1 water : DMSO cluster is formed for a stable CHS backbone network, including the hydrophobicity driven percolation effect^22^ and the CH/*n* HB effect. The CHS percolation model is depicted in Fig. 5a. A large HB network between DMSO–DMSO molecules emerges at the percolation threshold (6 : 1 water : DMSO) where the interlink of C–H⋯S forms the skeleton. Meanwhile, other HB interactions between DMSO and water are shown in Fig. 5b. Besides, the water shell model is suggested to depict the molecular cluster at higher water concentrations (Fig. 5c). The CHS model forms at 0–60 wt% while the excessive water molecules break the long-range connectivity between each DMSO molecule at 60–70 wt% and DMSO–water cluster transforms into the water shell model at 70–90 wt%.

The proposed model and mechanisms are in alignment with the abovementioned observations. Regarding the resonant frequency shift of C–H bending, the increased polarization of the C–H bond induced by the oxygen atom in water leads to an increase in the intramolecular attraction between the carbon and hydrogen atoms in DMSO (Fig. 1), surpassing the intermolecular attraction between DMSO and water. Consequently, the C–H bond distance in DMSO decreases, resulting in the blue shift of bending mode. As the C–H bonds of DMSO become saturated with HBs at a water concentration exceeding 70 wt%, water encapsulates the DMSO molecules, further shortening the C–H bond length due to the hydrophobic nature of methyl groups, thereby maintaining the resonant frequency blue shift.

Conversely, the blue shift of C–S stretching mode arises from two primary factors: the formation of a CH–water HB^10^ and CHS HB. Both factors contribute to the enhanced polarization of the C–S bond at low water percentages, saturating around ∼60 wt% with diminishing effects thereafter. An opposite influence is observed as the CHS HB breaks and the CH–water HB forms during the transition from CHS percolation to the water shell model, concluding at the 70 wt% mark with a discernible minimum point. Within the 70–90 wt% range, the blue shift of the resonant frequency is attributed to the encapsulation effect of the water shell.

Furthermore, alterations in vibrational dephasing time are examined. The organization of the DMSO–water cluster (Fig. 5a) prolongs the transient coherent polarization toward a high entropy, equilibrium state, resulting in increased dephasing times from 0 to 60 wt% water. The percolation threshold is measured to be at 61 wt% through the intersection of linear fittings. As the water concentration increases beyond 60 wt%, there are no factors sustaining the percolation state, leading to a decrease in dephasing time of the C–H bending mode. This indicates that the water shell creates a distinct environment for DMSO molecules to bend freely. Experiment findings underscore the sensitivity of measurements of molecular dynamics to subtle changes in local molecular interactions, particularly those involving vibrational modes with angular momentum, such as bending.

## Conclusions and outlook

In summary, we investigate the formation of CH/*n* HB networks in a binary aqueous system and, for the first time to our knowledge, demonstrate the measurement of the percolation threshold. Nonetheless, the observed resonant frequency shift of C–S stretching mode implies the potential formation of a sulfur atom-based backbone network. Vibrational dephasing represents a statistical decoherence process among ensemble molecular dipoles, necessitating a prolonged time to reach the equilibrium when the HB network restricts C–H bending to maintain bond angles within large clusters. Overall, these insights highlight the potential of T-CARS in identifying intricate molecular architectures upheld by CH/*n* HB interactions, with opportunities in the detection and characterization of HBs in various aqueous chemical and biological systems.

Beyond current findings on weak HB networks, there are several future directions to pursue. An interesting study is to develop applications for regulating protein functions through CH/*n* HB networks. Meanwhile, considering the energy aspect of vibrational dephasing,^42^ T-CARS is a preferable tool to investigate vibronic energy flow in protein percolation clusters, providing opportunities for assessing reactivities. Furthermore, future investigation of CH/*n* HB networks is proposed in aligned clusters, such as the phospholipid layer, for biology and materials science.

## Author contributions

Hanlin Zhu: experiment performance, data analysis, writing – original draft. Xinyu Deng: experiment performance, data analysis. Vladislav V. Yakovlev: supervision, funding acquisition, writing – review & editing. Delong Zhang: funding acquisition, resources, project administration, writing – review & editing.

## Conflicts of interest

There are no conflicts to declare.

## Supplementary Material

SC-015-D4SC03985H-s001

## Data Availability

Compound characterization data and experimental procedures are available in the ESI.†
