# Supplementary material for: Dynamics of CH/n hydrogen bond networks probed by time-resolved CARS spectroscopy
Source: Chem Sci. 2024 Jul 31;15(35):14344–51. doi: 10.1039/d4sc03985h (PMC11323804; doi:10.1039/d4sc03985h)
Supplement: SC-015-D4SC03985H-s001 [file SC-015-D4SC03985H-s001.pdf]

Supplementary Information (SI) for Chemical Science.
This journal is © The Royal Society of Chemistry 2024

ARTICLE

Dynamics of CH/n Hydrogen Bond Network Probed by Time-Resolved CARS Spectroscopy

Received 00th January 20xx,
Accepted 00th January 20xx

DOI: 10.1039/x0xx00000x

Hanlin Zhu†[a,b], Xinyu Deng†[a,b], Vladislav V. Yakovlev*[c,d,e], Delong Zhang*[a,b]

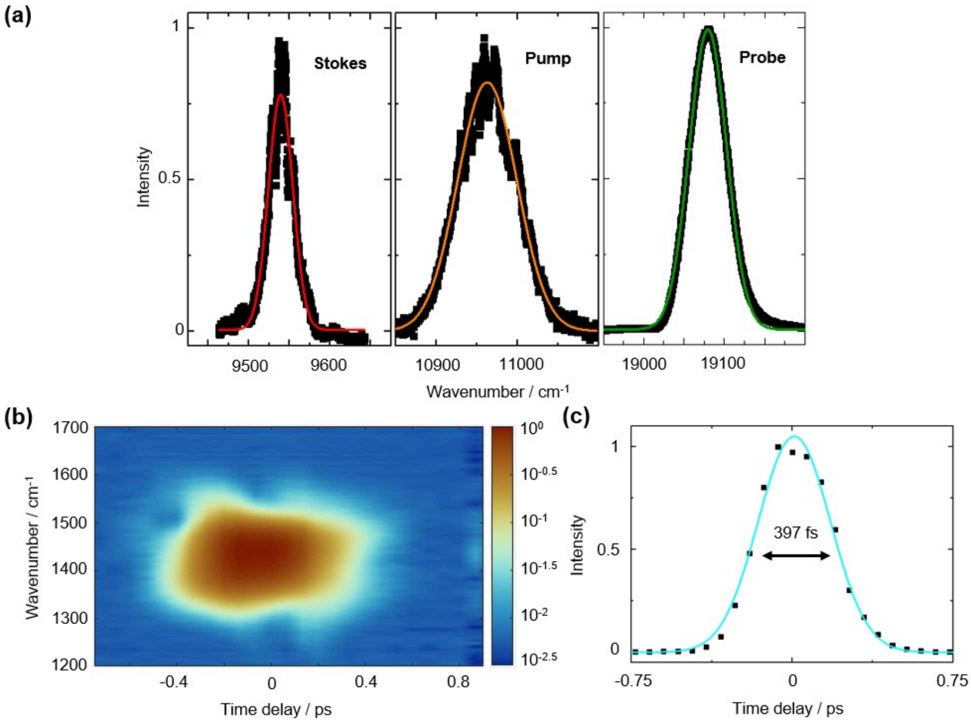

Fig. S1. Time-resolved coherent anti-Stokes Raman scattering (T-CARS) spectroscopy platform. (a) Representative spectra of the stimulation beams, Stokes, pump, and probe beams. Spectral bandwidth (FWHM) of each beam is 35 cm^{-1}, 86 cm^{-1}, and 55 cm^{-1}. (b) Time- frequency-resolved spectrum of water. The spectra are colored with the Roma color map. (c) Pulse width (FWHM) of four-wave-mixing signal of water.

[a] *Zhejiang Key Laboratory of Micro-nano Quantum Chips and Quantum Control, School of Physics, Zhejiang University, Hangzhou, Zhejiang 310027, China.*
[b] *MOE Frontier Science Center for Brain Science & Brain-Machine Integration, Zhejiang University, Hangzhou, Zhejiang 310027, China.*
[c] *Department of Biomedical Engineering, Texas A&M University, College Station, TX 77843, USA.*
[d] *Department of Physics and Astronomy, Texas A&M University, College Station, TX 77843, USA.*
[e] *Department of Electrical and Computer Engineering, Texas A&M University, College Station, TX 77843, USA.*
† These authors contributed equally to this work.
Supplementary Information available: [details of any supplementary information available should be included here]. See DOI: 10.1039/x0xx00000x

Please do not adjust margins

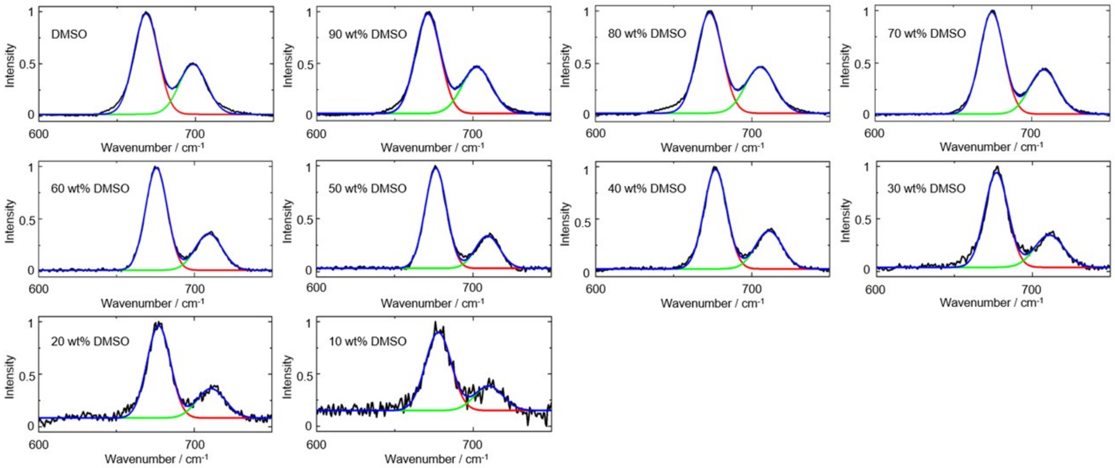

Fig. S2. Bimodal Gaussian fitting of C-S stretching symmetric and anti-symmetric modes.

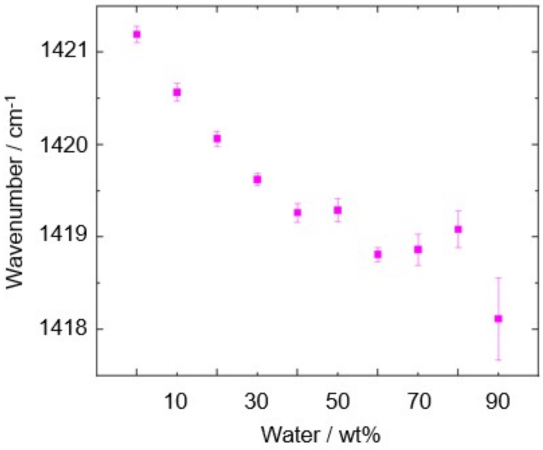

Fig. S3. Resonant frequency of C-H bending mode plotted against water concentration in weight percent.

 This journal is © The Royal Society of Chemistry 20xx

Please do not adjust margins

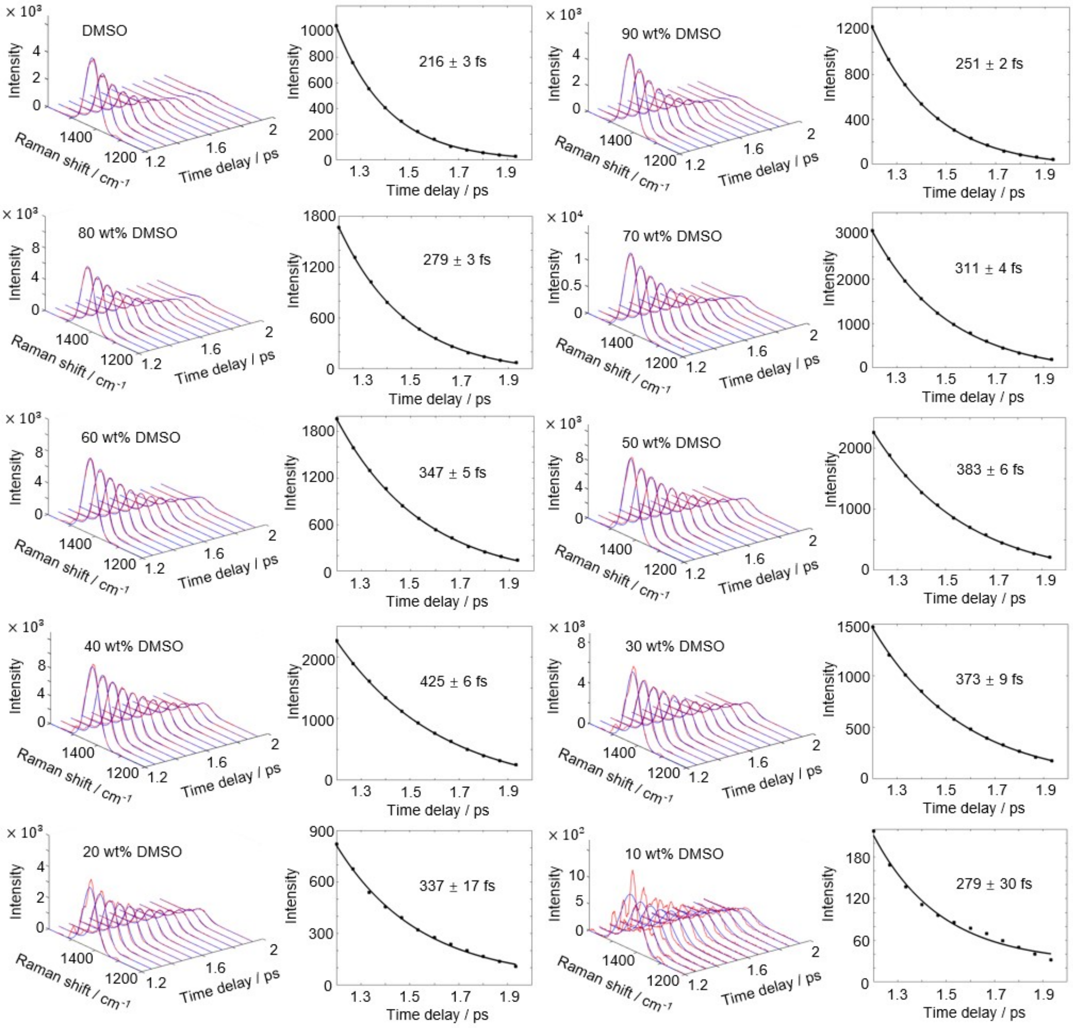

Fig. S4. T-CARS spectra of DMSO-water solution.
